# Investigating Glioblastoma Multiforme Sub-Proteomes: A Computational Study of CUSA Fluid Proteomic Data

**DOI:** 10.3390/ijms23042058

**Published:** 2022-02-12

**Authors:** Fabiana Moresi, Diana Valeria Rossetti, Federica Vincenzoni, Giorgia Antonia Simboli, Giuseppe La Rocca, Alessandro Olivi, Andrea Urbani, Giovanni Sabatino, Claudia Desiderio

**Affiliations:** 1 Department of Neurosurgery, Mater Olbia Hospital, 07026 Olbia, Italy; fabiana_moresi@libero.it (F.M.); giuseppe.larocca@policlinicogemelli.it (G.L.R.); giovanni.sabatino@policlinicogemelli.it (G.S.); 2 Dipartimento di Scienze Biotecnologiche di Base, Cliniche Intensivologiche e Perioperatorie, Università Cattolica del Sacro Cuore, 00168 Rome, Italy; federica.vincenzoni@unicatt.it (F.V.); andrea.urbani@unicatt.it (A.U.); 3 Istituto di Scienze e Tecnologie Chimiche “Giulio Natta”, Consiglio Nazionale delle Ricerche, 00168 Rome, Italy; dianavaleria.rossetti1@unicatt.it; 4 Fondazione Policlinico Universitario A. Gemelli IRCCS, 00168 Rome, Italy; giorgia.simboli573@gmail.com (G.A.S.); alessandro.olivi@policlinicogemelli.it (A.O.); 5 Institute of Neurosurgery, Fondazione Policlinico Universitario A. Gemelli IRCCS, Catholic University, 00168 Rome, Italy

**Keywords:** brain tumor, glioblastoma multiforme, CUSA fluid, proteomics

## Abstract

Based on our previous proteomic study on Cavitating Ultrasound Aspirator (CUSA) fluid pools of Newly Diagnosed (ND) and Recurrent (R) glioblastomas (GBMs) of tumor core and periphery, as defined by 5-aminolevulinc acid (5-ALA) metabolite fluorescence, this work aims to apply a bioinformatic approach to investigate specifically into three sub-proteomes, i.e., Not Detected in Brain (NB), Cancer Related (CR) and Extracellular Vesicles (EVs) proteins following selected database classification. The study of these yet unexplored specific datasets aims to understand the high infiltration capability and relapse rate that characterizes this aggressive brain cancer. Out of the 587 proteins highly confidently identified in GBM CUSA pools, 53 proteins were classified as NB. Their gene ontology (GO) analysis showed the over-representation of blood coagulation and plasminogen activating cascade pathways, possibly compatible with Blood Brain Barrier damage in tumor disease and surgery bleeding. However, the NB group also included non-blood proteins and, specifically, histones correlated with oncogenesis. Concerning CR proteins, 159 proteins were found in the characterized GBM proteome. Their GO analysis highlighted the over-representation of many pathways, primarily glycolysis. Interestingly, while CR proteins were identified in ND-GBM exclusively in the tumor zones (fluorescence positive core and periphery zones) as predictable, conversely, in R-GBM they were unexpectedly characterized prevalently in the healthy zone (fluorescence negative tumor periphery). Relative to EVs protein classification, 60 proteins were found. EVs are over-released in tumor disease and are important in the transport of biological macromolecules. Furthermore, the presence of EVs in numerous body fluids makes them a possible low-invasive source of brain tumor biomarkers to be investigated. These results give new hints on the molecular features of GBM in trying to understand its aggressive behavior and open to more in-depth investigations to disclose potential disease biomarkers.

## 1. Introduction

Glioblastoma multiforme (GBM) is the most frequent brain tumor in adults and is classified as WHO grade IV tumor for its high grade of malignancy [1]. Despite the continuous research for new molecular targeted therapies, the outcome for patients is still fatal, because of the extraordinary therapeutic resistance and the recurrence phenomena after surgical removal. Its molecular features and pathologic profile are still far from being fully clarified. Progress in the comprehension of these characteristics is necessary to develop new molecular targeted therapies [2].

Proteomics represents an analytical tool that, as well as all other “omic” sciences and in complement with them, plays an important role in the definition of the molecular basis underlying the onset and the progression of diseases. Clinical proteomics specifically aims to characterize the proteins and peptides expressed by a cell, a tissue or a biological fluid, and to study their alterations in pathological conditions [3]. In previous work we analyzed the proteomic profile of Cavitating Ultrasound Aspirator (CUSA) fluid from seven patients affected by Newly Diagnosed (ND) and Recurrent (R) GBM. The samples were collected from different zones of the tumor, according to the results obtained after administration of 5-aminolevulinic acid (A). This acid is the initiator of porphyrins’s biosynthesis and it is used in surgery to identify the tumor zone thanks to the capability of the tumor cells to produce the fluorescent protoporphyrin IX metabolite from this acid [4]. Although the acid is not directly responsible for the fluorescence, by convention we used the following acronyms to distinguish the different tumor peripheral zones of collection, i.e., the fluorescence positive (A+) and negative (A−) tumor periphery with respect to the tumor core (CORE). Moreover, it was possible to define a “tumor zone”, enclosing the tumor core and the A+ periphery, and the “healthy zone”, i.e., the A− peripheral zone, and to characterize their differential protein profiles [1]. The present investigation aims to deeply explore these proteomic data by applying a bioinformatic approach to specifically investigate three sub-proteomes, including the Not Detected in Brain (NB), Cancer Related (CR) and Extracellular Vesicles (EVs) protein classes, following the Human Protein Atlas classification [5], and to compare their distribution inside the different tumor zones and the different GBM diagnosis, i.e., newly diagnosed tumor and relapse.

The choice to investigate these groups of proteins arises from (i) the interest in distinguishing brain from Non-Brain protein elements in the GBM proteome, using as reference the list of proteins classified in the Human Protein Atlas as “Proteins Not Detected in Brain”, (ii) the purpose to deeply investigate and classify the Cancer Related protein elements in the GBM proteome using as reference the Human Protein Atlas list of “Cancer Related proteins”; and (iii) the rationale to evaluate the EVs protein components in the GBM proteome, using as reference the Human Protein Atlas “Extracellular Vesicles proteins” class, due to the role of EVs in cancer and tumor diffusion of growing interest of investigation in the recent years [6]. The availability of data from different zones of collection allowed also to interestingly investigate the specific distribution of these protein classes in the “tumor” as well as in the “healthy zone” of ND- and R-GBM diagnosis. All together the study of each of these specific protein classes encompasses the common purpose to add new hints for the understanding of the molecular features at the basis of the high diffusion and recurrence rate of GBM, as well as to disclose new potential biomarkers for clinical applications and early tumor diagnosis.

## 2. Materials and Methods

### 2.1. Chemicals

5-aminolevulinic acid (5-ALA) was from Medac (Wedel, Germany). Iodoacetamide (IAA), D,L-dithiothreitol (DTT), Ammonium bicarbonate (AMBIC), bovine serum albumin and acetone were from Sigma-Aldrich (St. Louis, MO, USA). Water, acetonitrile (ACN), formic acid (FA) were from Merck (Darmstadt, Germany). All organic solvents were of LC-MS grade. Trypsin enzyme (Gold MS Grade) was from Promega (Madison, WI, USA).

### 2.2. Bioinformatic Data Elaboration

LC-MS and MS/MS data were elaborated by Proteome Discoverer 1.4 software (version 1.4.1.14, Thermo Fisher Scientific), based on SEQUEST HT cluster as search engine against the Swiss-Prot Homo Sapiens proteome as described in detail in a previous paper [1].

Briefly, the following parameters were entered for the analysis:

- Minimum precursor mass 350 Da;
- Maximum precursor mass 10,000 Da;
- Total intensity threshold 0.0;
- Minimum peak count 1;
- Signal to Noise (S/N) threshold 1.5;
- Mass tolerance 10 ppm;
- Fragment mass tolerance 0.5 Da;
- Use average precursor mass False;
- Use average fragment mass False;
- Trypsin enzyme with a maximum of 2 missed cleavage sites;
- The minimum and maximum peptide length was 6 and 144 residues, respectively;
- Dynamic methionine oxidation (+15.99 Da);
- Static carbamidomethylation of cysteine (+57.02 Da).

Protein and peptide spectra matches were validated by the calculation of false discovery rate (FDR) using the Percolator node. The strict target FDR value was set at 0.01, while the relaxed value was set at 0.05. Protein identification results were further filtered according to the Human Proteome Project Mass Spectrometry Data Interpretation Guidelines [7] for high peptide confidence identification:

- Peptide confidence high;
- Minimum peptide length ≥ 9 amino acid residues;
- Peptide rank 1;
- Minimum 2 peptides per protein.

**Figure 1:**
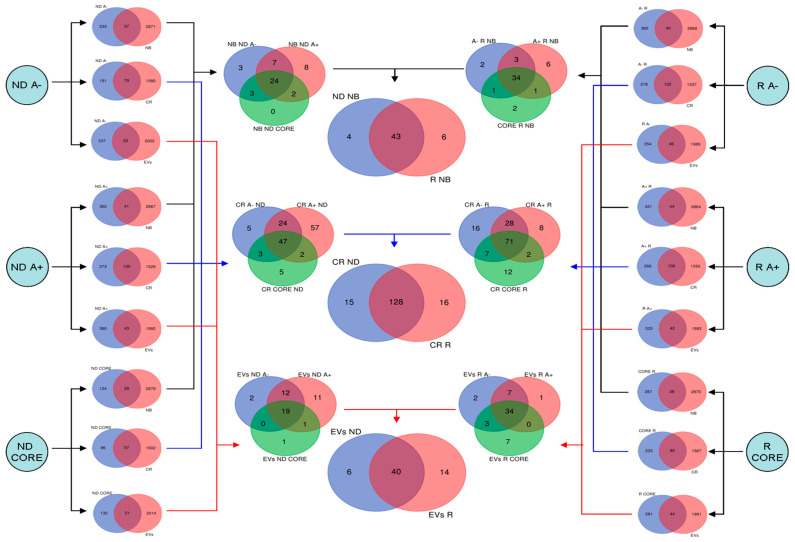
Workflow applied for the computational study of GBM sub-proteomes based on different tumor zones, i.e., CORE, A+ and A−, and GBM diagnosis, i.e., ND- and R-GBMs.

The lists of proteins identified in our previous study in the six pools of different tumor zones and GBM diagnosis, i.e., ND CORE, A+ and A−, and R CORE, A+ and A− obtained by seven patients [1], have been further analyzed by bioinformatic approach to investigate three different specific protein classes, following the Human Protein Atlas [5] classification: (i) Proteins Not Detected in Brain (NB), which count 2908 elements; (ii) Cancer Related proteins (CR), which count 1659 elements; (iii) Extracellular Vesicles (EVs) proteins, which count 2035 elements. It is here specified that only the protein elements with Uniprot accession have been considered for the study and the bioinformatic elaboration, by applying the following workflow (Figure 1):

- Venn diagrams [8] were elaborated to group the GBM protein elements of each pool with respect to the NB, CR and EVs protein class lists available in the Human Protein Atlas [5]; in this way, for each pool it was possible to classify the members of each protein class studied.
- The elements of each protein class found in each pool were further grouped by Venn diagram construction to define for each sub-proteome the common and unique elements of each zone, i.e., CORE, A+ and A−, for both ND- and R-GBMs.
- Finally, the obtained lists of proteins belonging to the NB, CR and EVs sub-proteomes of ND- and R-GBMs were compared to distinguish the common and the exclusive elements of each GBM diagnosis.

Gene Ontology (GO) classification and pathways over-representation analysis were performed by Protein ANalysis THrough Evolutionary Relationships (PANTHER, http://www.pantherdb.org, accessed on 4 February 2022) Classification System (version 16.0) [9] using Fisher’s Exact test type and correction of false discovery rate (FDR). Protein–protein functional interaction networks were investigated through STRING database [10].

Significant differences in protein levels between samples, obtained by label free relative quantization, considering the average protein area values of three analytical replicates, were calculated by one-way ANOVA with Tukey’s post hoc test and considering *p*-values < 0.05 as significant.

## 3. Results

The lists of proteins identified in CUSA fluid of Newly Diagnosed (ND) and Recurrent (R) GBM pools collected in different tumor zones by LC-MS analysis following a shotgun proteomic approach [1] are here the object of a computational study to specifically investigate the presence of elements belonging to the Non-Brain, Cancer Related and EVs protein classes, following the Protein Atlas classification of reference [5], and to study their distribution per tumor zones and diagnosis. These protein classes are of great interest in studying cancer diseases since they are involved in oncogenesis and tumor diffusion, i.e., Cancer Related and EVs proteins, or possibly disclose uninvestigated molecular mechanisms, i.e., elements of Non-Brain protein class unexpectedly characterized in brain tumor tissue and proximate brain tissue periphery. The Human Protein Atlas was used as the database of reference for the classification of these groups of proteins included under the name of “Proteins Not Detected in Brain”, “Cancer Related proteins”, and “Extracellular Vesicles proteins” classes. The availability of data from CUSA fluid samples collected in different zones from the tumor core to the healthy zone at tumor periphery allowed to interestingly investigate the specific distribution of these protein classes in the tumor as well as in the healthy zone of ND- and R-GBM pools.

Considering both pools, a total of 587 proteins were identified by LC-ESI-MS/MS and, out of these, 270, 430 and 153 elements were identified in A−, A+ and CORE of ND-GBM and 400, 375 and 325 in A−, A+ and CORE of R-GBM, respectively [1]. These data were analyzed following the workflow illustrated in Figure 1 and described in detail in the Materials and Methods section. The next paragraphs separately describe the results obtained for each protein class analyzed followed by the relative discussion.

### 3.1. Proteins Not Detected in Brain (NB)

According to the workflow in Figure 1, by comparing the lists of protein elements identified in CUSA fluids samples of ND- and R-GBMs [1] with the sub-proteome class of “Proteins Not Detected in Brain” (NB) available in the Human Protein Atlas [5], 37 elements in A− ND, 41 in A+ ND, 29 in CORE ND, 40 in A− R, 44 in A+ R and 38 in CORE R resulted as NB proteins.

**Figure 2:**
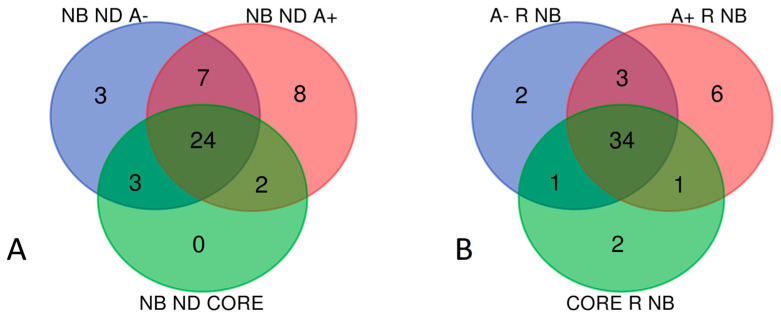
(**A**) NB proteins found in ND-GBM pool, (**B**) NB proteins found in R-GBM pool and the relative zone of identifications.

Grouping analysis by Venn diagram elaboration showed 47 unique NB elements in ND-GBM considering both shared and exclusive elements of the three analyzed zones: specifically, 24 proteins were commonly identified in CORE, A+ and A− zones, while the A− and A+ zones showed 3 and 8 exclusive elements, respectively, 7 proteins were shared by both, and no CORE exclusive elements were found (Figure 2A). The same data analysis was applied to the R-GBM list of proteins, resulting in 49 NB unique elements, 34 shared by all zones, with few elements exclusive to each zone and corresponding to 2, 6 and 2 NB proteins for the CORE, the A+ and the A− zones, respectively (Figure 2B).

**Figure 3:**
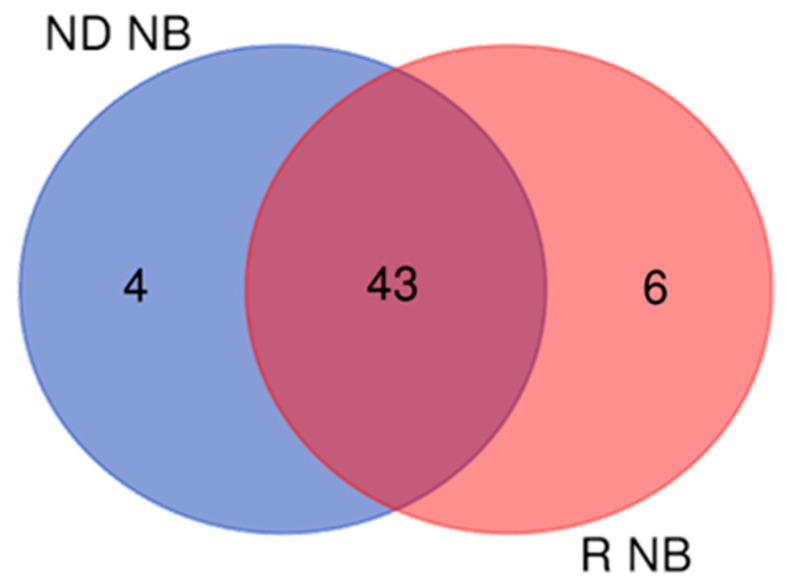
Venn diagram between Newly Diagnosed (ND) and Recurrent (R) non-brain (NB) proteins: 43 are common to both GBM pools, 4 and 6 are exclusive of ND- and R-GBM, respectively.

Comparing the 47 total NB proteins of ND-GBM with the 49 total NB proteins of R-GBM, grouping analysis results in 53 unique protein elements distributed as follows: 43 proteins resulted in being common to both GBMs, while 4 and 6 NB proteins were exclusive to ND- and R-GBMs, respectively (Figure 3). Table 1 and Table 2 list the protein elements exclusive to ND- and R-GBMs, respectively, also describing the zone of identification. The list and zone of identification of the 43 common elements is reported in Supplementary Table S1.

Interestingly, the group of proteins exclusive to ND- and of R-GBMs are all localized in the tumor zone (A+ and/or tumor CORE), with the exception of complement factor H-related protein 1 being also present in the A− zone of R-GBM, and they could therefore potentially distinguish newly diagnosed tumor from relapse.

**Figure 4:**
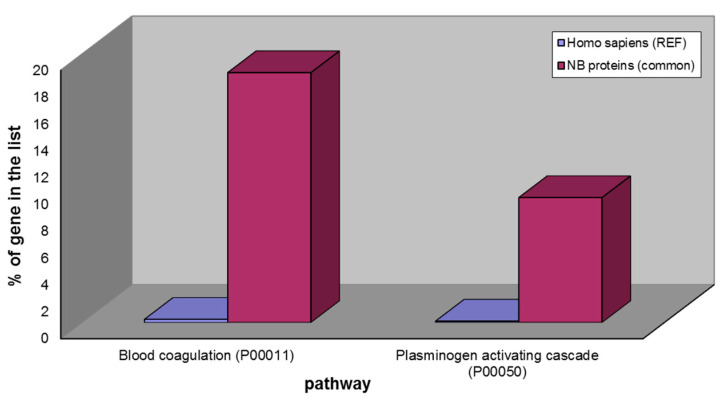
Over-represented pathways of the 43 NB proteins common to ND- and R-GBM pools: blood coagulation and plasminogen activating cascade.

Considering the 43 common elements of ND- and R-GBMs instead, the GO analysis by the PANTHER tool [9] showed the over-representation of two pathways with respect to the human database of reference, namely, the blood coagulation and the plasminogen activating cascade pathways (Figure 4) that could be explained by Blood Brain Barrier (BBB) damage generally occurring in brain tumor diseases and, as expected, by bleeding during surgery. 

**Figure 5:**
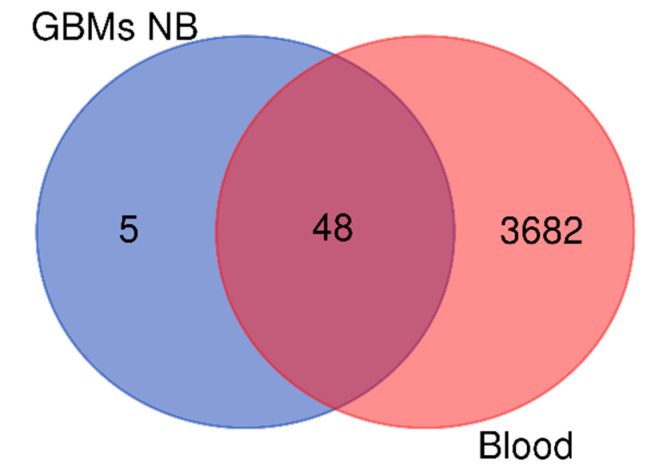
Venn diagram of the 53 NB proteins of the samples and blood proteins of Human Protein Atlas.

Therefore, to evaluate the contribution of Blood Brain Barrier damage and bleeding to NB protein enrichment in GBM CUSA fluid, the total list of the 53 NB unique elements identified in GBM (Figure 3) was compared with the list of blood proteins class available in the Human Protein Atlas [5] (Figure 5). With the exception of five elements, all the NB proteins found in GBM were classified as blood proteins.

Focusing the attention on the five non-blood proteins, it is worthy of mention that they include four histones, highly basic proteins correlated with the onset of several tumors [11], and the serum amyloid A1 protein, an acute-phase inflammation protein upregulated in human glioblastoma [12]. Table 3 reports the protein name and the Uniprot accession of the five non-blood NB elements and their zone of identification.

Interestingly, the serum amyloid A-1 protein exclusively marked the tumor zone of R-GBM, while all the other elements were characterized in both the GBM diagnoses. Histone H2A type 1-H and H4 were identified in the same zones in both the GBM pools, therefore not exhibiting any apparent interesting differences between ND- and R-GBM. Histone H2A type 1-B/E was instead identified in the ND-GBM healthy zone (A− zone) and in the tumor CORE of R-GBM, this result being particularly interesting to investigate as this protein was characterized in opposite zones of the two tumor diagnoses.

**Figure 6:**
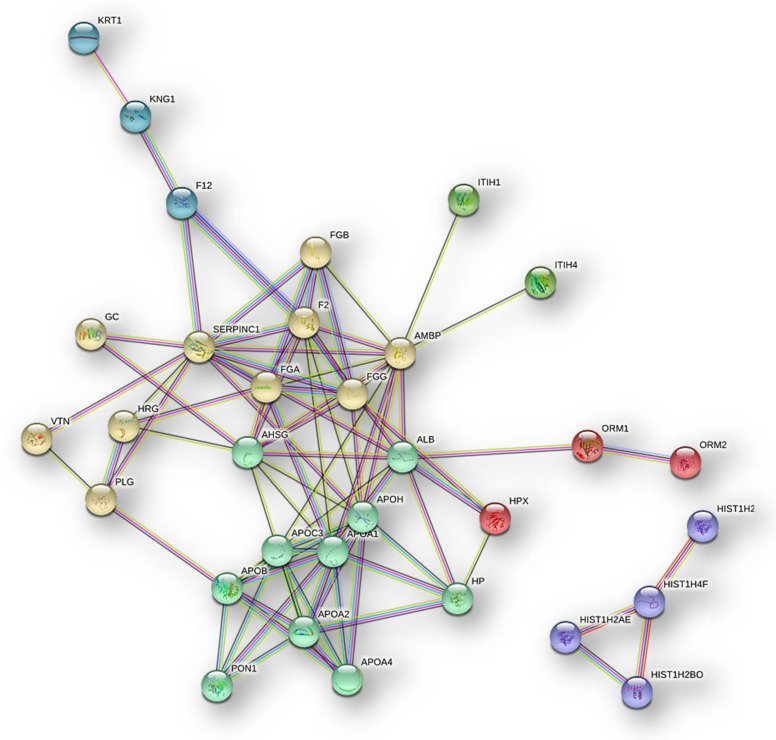
Protein–protein functional interaction network (highest confidence) and clusters of the 43 NB proteins common to Newly Diagnosed and Recurrent GBM pools.

The further analysis of the 43 NB proteins common to ND- and R-GBM pools (Figure 3) for investigating the functional interactions by STRING bioinformatics tool [10] showed, in highest confidence, a dense network of interactions (Figure 6) and evidenced six clusters, hiding the disconnected nodes.

### 3.2. Cancer Related Proteins (CR)

**Figure 7:**
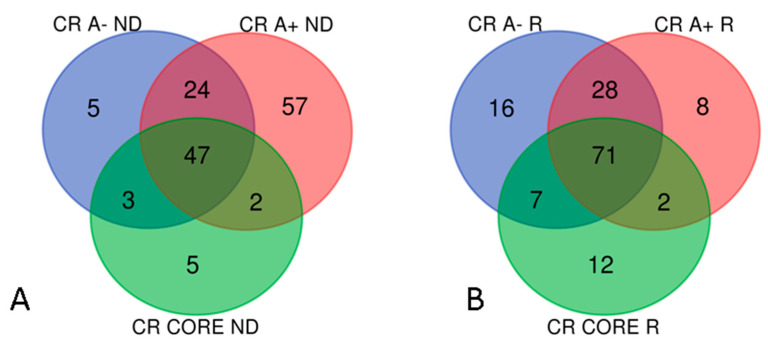
Grouping analyses of the CR proteins found in ND-GBM (**A**) and R-GBM (**B**) pools and their zone of identification.

According to the workflow in Figure 1, comparing the proteins lists resulting from the LC-ESI-MS/MS analysis with the sub-proteome of “Cancer Related Proteins” (CR) available in the Human Protein Atlas [5], 79 elements in A− ND, 130 in A+ ND, 57 in CORE ND, 122 in A− R, 109 in A+ R and 92 in CORE R resulted as CR proteins. Venn diagram grouping of all the ND-GBM Cancer Related proteins resulted in 143 unique elements shared and exclusively distributed in the diverse zones as in Figure 7A. The same analysis was performed for the R-GBM data, resulting in 144 unique elements grouped in the diverse zones as described in Figure 7B.

**Figure 8:**
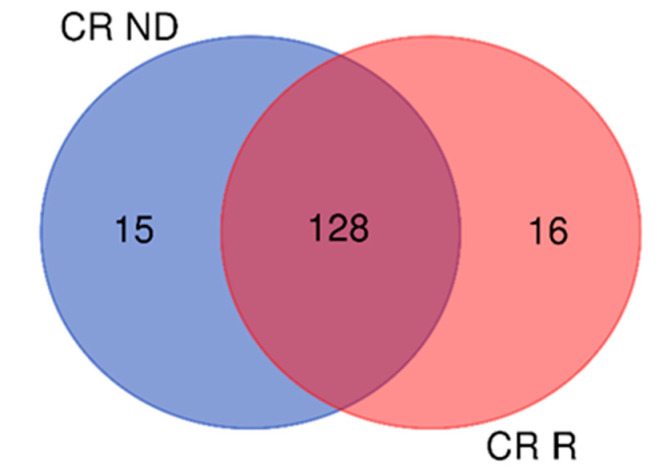
Venn diagram of CR proteins of the samples: 128 elements were common to both GBM pools, while 15 and 16 were typical of ND- and R-GBMs, respectively.

Comparing the 143 CR ND-GBM proteins with the 144 CR R-GBM proteins, 128 elements were shared by both GBMs, while 15 and 16 protein elements were exclusive to ND- and R-GBMs, respectively, as resulting from Venn diagram in Figure 8. Table S2 in the Supplementary Materials lists the 128 CR proteins commonly identified in ND- and R-GBM pools, also reporting the relative zone of identification.

The exclusive proteins potentially distinguishing ND- from R-GBMs are listed in Table 4 and in Table 5 with the indication of the zone of characterization. It is noteworthy that also for this class of proteins, the exclusive proteins of ND-GBM have been all detected in the tumor zone. In R-GBM some of the exclusive proteins were instead found distributed also in the healthy A− zone.

**Figure 9:**
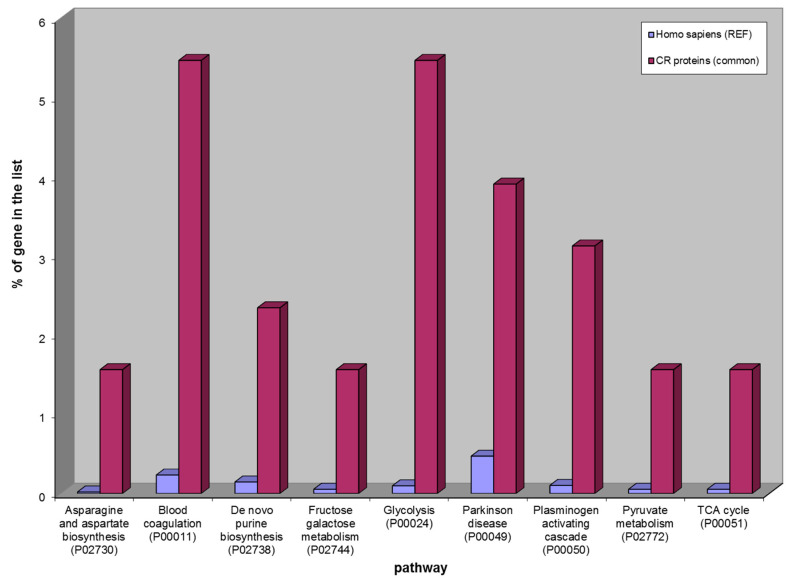
Over-represented pathways of 128 CR proteins common to Newly Diagnosed and Recurrent GBMs. From the left: asparagine and aspartate biosynthesis, blood coagulation, de novo purine biosynthesis, fructose galactose metabolism, glycolysis, Parkinson disease, plasminogen activating cascade, pyruvate metabolism, TCA cycle.

The Gene Ontology analysis of the 128 CR proteins identified in both ND- and R-GBM pools highlighted that, in addition to the already mentioned blood coagulation and plasminogen activating cascade, other pathways resulted as being over-represented, namely, asparagine and aspartate biosynthesis, de novo purine biosynthesis, fructose galactose metabolism, glycolysis, Parkinson disease, pyruvate metabolism and TCA cycle (Figure 9).

**Figure 10:**
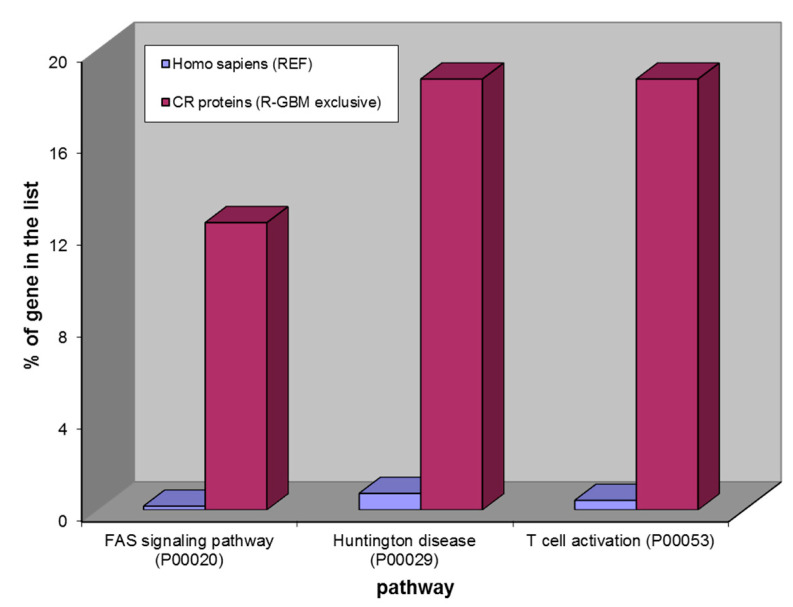
Over-represented pathways of 16 CR proteins exclusively found in Recurrent GBM. From the left: FAS signaling pathway, Huntington disease and T cell activation.

The same analysis was performed on the CR proteins exclusive to ND- and R-GBMs. The over-represented pathways analysis of the 15 ND-GBM exclusive elements (Figure 8) provided the same results of the 128 common CR proteins (Figure 9). Instead, the over-represented pathways of the 16 CR proteins exclusive to R-GBM were different, as shown in Figure 10. Indeed, three different pathways resulted as being over-represented, namely, the FAS signaling pathway, Huntington disease and T cell activation pathways, that therefore seem involved in tumor relapse, differently from GBM of new diagnosis.

**Figure 11:**
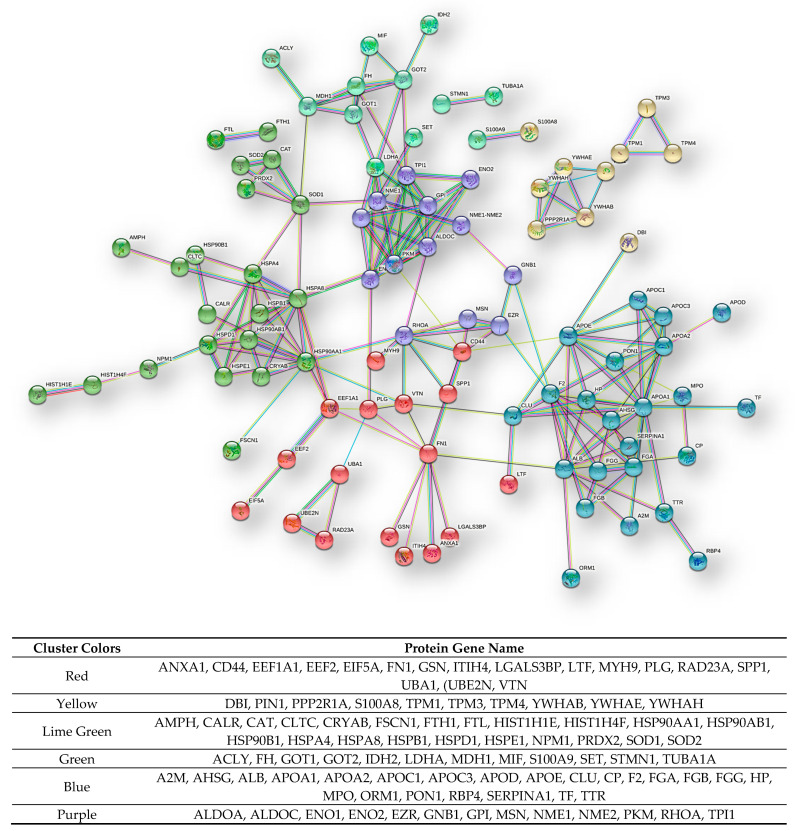
STRING functional interaction network (highest confidence) and clusters of the 128 CR proteins common to both GBMs pools. At the bottom the list of the gene names of the proteins enclosed in the different clusters is reported.

The bioinformatics data elaboration of the protein–protein interactions relative to the 128 proteins common to ND- and R-GBMs by STRING tool [10] showed, with highest confidence, a dense network of interactions with the disconnected nodes hidden, evidencing six clusters (Figure 11).

### 3.3. Extracellular Vesicles Proteins (EVs)

**Figure 12:**
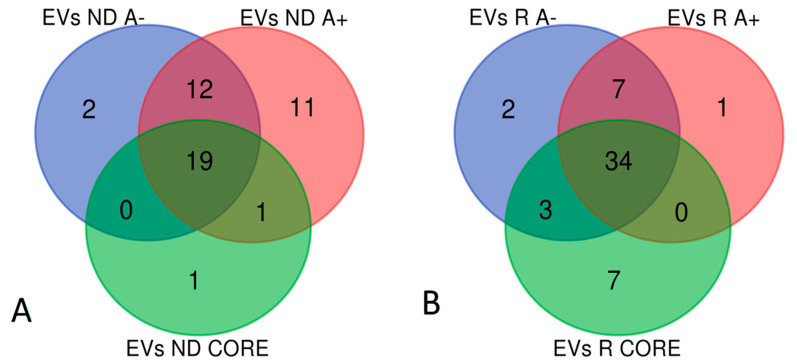
Grouping analyses of the EVs proteins found in ND-GBM (**A**) and in R-GBM (**B**) pools and the relative zone of identification.

According to the workflow in Figure 1, comparing the lists of elements resulting from the LC-ESI-MS/MS analysis with the sub-proteome class of “Extracellular Vesicles proteins” (EVs) available in the Human Protein Atlas [5], 33 elements in A− ND, 43 in A+ ND, 21 in CORE ND, 46 in A− R, 42 in A+ R and 44 in CORE R resulted as EVs proteins. Grouping all the EVs ND-GBM proteins found, avoiding the redundant elements, resulted in 46 unique elements. The Venn diagram in Figure 12A shows their distribution within the diverse zones. The same analysis was performed for the EVs R-GBM proteins, resulting in 54 unique elements (Figure 12B).

**Figure 13:**
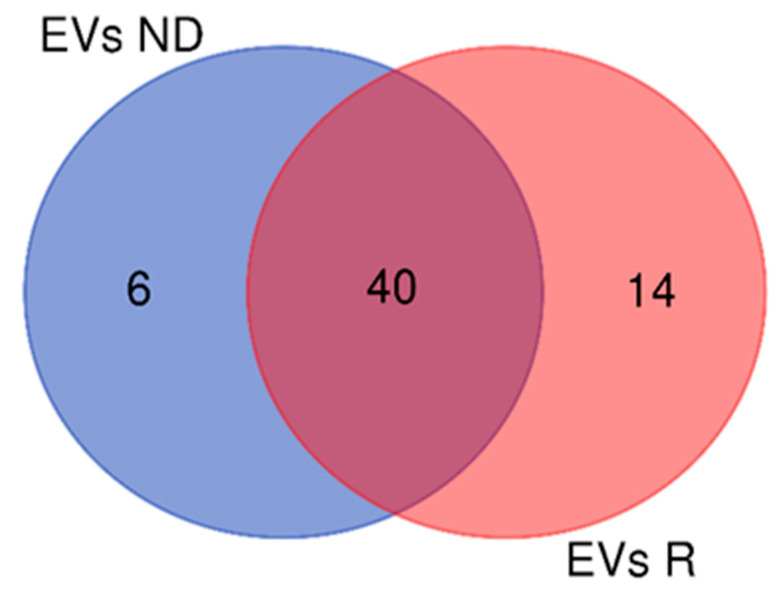
Venn diagram of EVs proteins of the samples: 40 were present in both ND- and R-GBM pools; 6 only in ND-GBMs and 14 in R-GBMs.

Comparing the 46 EVs proteins of ND-GBM with the 54 EVs proteins of R-GBM, 40 elements were shared by both, while 6 and 14 proteins were exclusive to ND- and R-GBM pools, respectively (Figure 13). Table S3 in Supplementary Materials describes the EVs elements commonly identified in ND- and R-GBM pools and the relative zones of identification. Table 6 and Table 7 list the EVs proteins exclusively marking the ND- and R-GBMs, also reporting the relative zone of identification.

**Figure 14:**
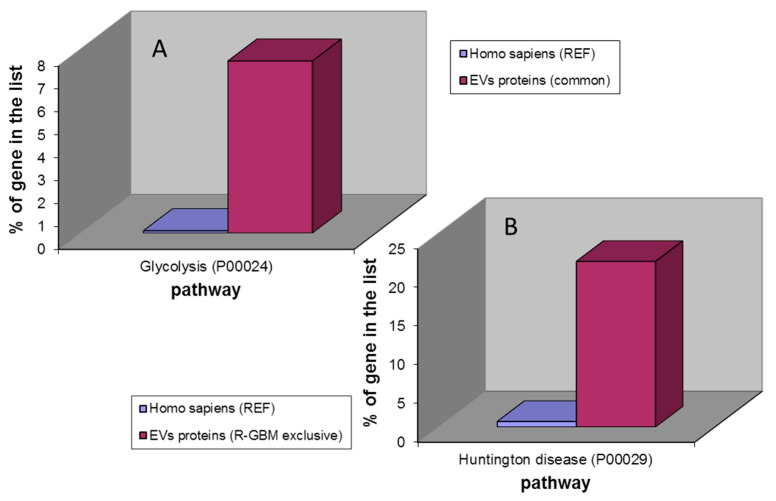
(**A**) Results of the PANTHER pathway over-representation analysis of 40 EVs common proteins to ND- and R-GBM pools (glycolysis) and (**B**) of 14 EVs proteins exclusive to R-GBM pool (Huntington disease).

The Gene Ontology analysis of the 40 EVs proteins common to ND- and R-GBMs showed only the glycolysis as the unique over-represented pathways (Figure 14A). The same analysis of the EVs proteins exclusive to ND-GBM did not show over-represented pathways, the limited number of elements to be taken into account, while the 14 EVs elements exclusive to R-GBM proved the over-representation of the Huntington disease pathway (Figure 14B).

**Figure 15:**
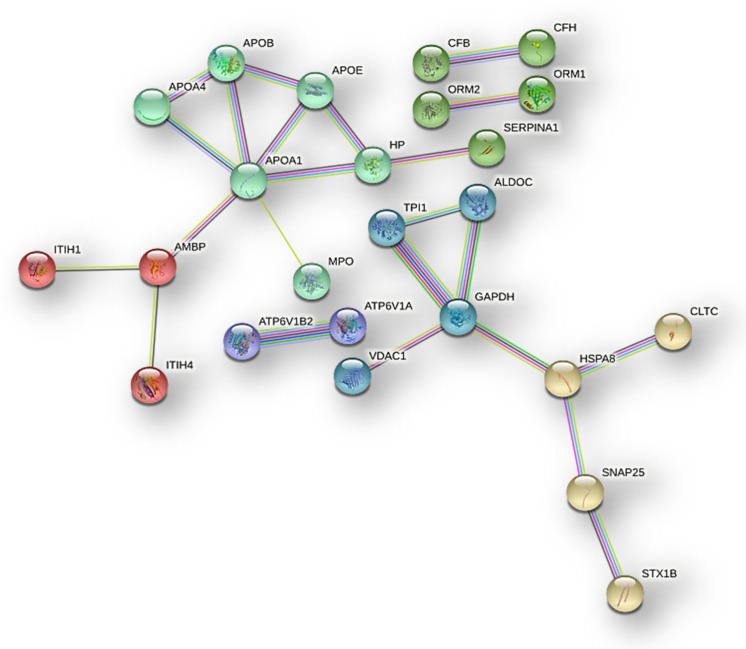
STRING tool analysis (highest confidence) of protein–protein interaction and clusters of the 40 EVs proteins found in Newly Diagnosed and Recurrent GBMs.

Moreover, STRING tool analysis [10] showed, with highest confidence, a deep interaction network of the common ND- and R-GBM EVs elements (Figure 15), in which it was possible to identify six clusters, hiding the disconnected nodes.

## 4. Discussion

### 4.1. Proteins Not Detected in Brain (NB)

From the results obtained it is worthy of mention that 53 proteins out of the total proteins identified in GBM samples cannot be classified as brain proteins, following the Protein Atlas database enclosing them in the list of Non-Brain proteins (Figure 3). All of them are involved in blood coagulation and plasminogen activating cascade pathways (Figure 4), in accordance with their inclusion in the list of proteins classified as blood proteins (Figure 5), with the exception of four histones and the serum amyloid A1 protein (Table 3) resulting Non-Brain and non-blood proteins. The very well-known biological role of histones is the packaging of chromatin. In pathological conditions, such as in tumor disease, they can be subjected to post-translational modifications (PTMs), such as acetylation, methylation, citrullination and ribosylation that differently modulate their functions [11]. Particularly interesting is the acetylation PTM, that influences several cellular processes. Indeed, a recent study hypothesized that histones’ acetylation, and particularly acetylation of histone H4, is relevant for the regulation of intracellular pH (pHi) and stated that tumor cells show pHi lower than normal cells [13]. Moreover, acetylation of histones is a way of regulation of genes’ transcription: in fact, in cancer cells the proto-oncogenes are hyperacetylated, resulting in stimulation of their transcription process. On the opposite side, tumor suppressor genes are hypoacetylated and their transcription is inhibited [14]. For this reason, histone H4 is enclosed in the CR class of proteins in addition to the NB protein class. It can be reasonably hypothesized that the presence in the brain of these kinds of proteins can be explained from the presence of the tumor itself.

Serum amyloid A1 (SAA1) is instead produced by the liver as an element of acute inflammation. Involved in the production of nitric oxide and other reactive oxygen elements, SAA1 seems to have a role in proliferation, migration, and invasion of cancer cells, including GBM cells [12]. Despite that its role as a prognosis biomarker has been validated in various types of cancer (such as melanoma, renal cell carcinoma and gastric, lung, breast and uterine cervical cancers), relative to GBM this role has yet to be confirmed [12].

The NB proteins exclusive to ND- and R-GBMs have been all identified in the tumor zone, A+ and/or CORE zones. Among them the beta-actin (ND-GBM specific element) and the serum amyloid A4 (R-GBM specific element) have been reported to have a role in the onset of glioblastoma. Beta-actin, a skeletal protein involved in cell motility, cell division and immune response processes, showed increased levels in several types of cancers including GBM [15]. Serum amyloid A4 (SAA4) shares the same features and functions of the SAA1 isoform, above discussed [16]. These proteins could be therefore candidate biomarkers to discriminate Newly Diagnosed from Recurrent GBM in the 5-ALA positive periphery zone.

### 4.2. Cancer Related Proteins (CR)

A total of 159 protein elements identified in GBM were classified as CR proteins (Figure 8). Although their finding was expected, their distribution within the pools and the diverse zones showed interesting results. To the best of our knowledge, some CR proteins exclusively identified in ND- or R-GBMs were identified for the first time in GBM even if their role in cancer disease is validated. The evidence of their presence in GBM could give new hints to the research of diagnostic or therapeutic biomarkers never investigated before.

Table 8 and Table 9 describe the functions of the CR proteins exclusively identified in ND- or R-GBMs, respectively, with gene name and specifying their previous identification in GBM disease. Moreover, while all the CR proteins that exclusively marked ND-GBM were identified, as expected, in the tumor zone (i.e., tumor core and A+ zone), on the contrary, in R-GBM the majority of them were identified in the healthy zone (A− tumor peripheral zone). 

This evidence confirmed our previous findings [1] and could be correlated with the high tumor aggressiveness, addressing the concept that what we think and see as “healthy tissue” already contains a tumor infiltration.

### 4.3. Extracellular Vesicles Proteins (EVs) 

EVs are very important in tumor disease because they release components in extracellular matrix (ECM) and in neighbor cells, inducing phenotypic modification and promoting, particularly in brain tumors, cerebral dysfunctions [6]. EVs can therefore influence the tumor microenvironment in two ways: either modifying ECM (releasing proteases, causing the matrix break up or promoting angiogenesis) or altering the gene expression (stimulating the transcription of oncogenes, promoting metastasis or realizing miRNA and lncRNA). A total of 60 proteins identified in GBM were classified as EVs proteins (Figure 13), 3 out of them (Table 10) already correlated to GBM and generally to brain tumors [46,47].

Glyceraldehyde-3-phosphate dehydrogenase (G3P) and prosaposin (SAP) have been correlated to the invasiveness of GBM [46], while Chitinase-3-like protein 1 (CH3L1) to a decreased survival rate [47]. G3P is the enzyme that catalyzes phosphorylation and oxidation of glyceraldehyde-3-phosphate to 1,3 bi-phosphoglycerate, using NAD+. This protein exerts, however, additional biological functions inside endocytosis, nuclear membrane assembly and cytoskeleton dynamic processes. G3P is also an RNA binding protein and interacts with the nucleic acids. Finally, it is one of the more important pro-apoptotic proteins. Despite its pro-apoptotic function, in tumors it results as being overexpressed. Its pathological features involve the rising of glycolysis, mitosis and ATP production processes, as well as the releasing of cytokines involved in the tumor progression and the DNA repair after damage caused by drugs [48].

SAP is a glycoprotein involved in metabolism of sphingomyelin and ceramide and in neuroprotection (repairs and regenerates neurons). In the presence of tumors, SAP results as being overexpressed and is associated with poor prognosis. Particularly, SAP binds TLR4, a membrane receptor, activating the transcription of the Myd88 gene, which induces the NF-kB pathways. This pathway promotes tumor growth, inducing inflammation [49].

CH3L1 is a member of glycoside hydrolases, with many physiological functions: stimulation of cell growth and proliferation, activation and differentiation of immune system and regulation of synthesis and degradation of ECM. CH3L1 is also a pro-inflammatory and anti-apoptotic factor. In tumors this protein is overexpressed, and it is involved in numerous functions, such as the promoting of tumor growth, proliferation, invasion and metastasis. It also has a role in angiogenesis and tumor inflammation [25]. For this reason, CH3L1 is present also in the list of Cancer Related proteins detected only in Newly Diagnosed GBM.

### 4.4. Protein Elements Sharing NB, CR and EVs Classification

**Figure 16:**
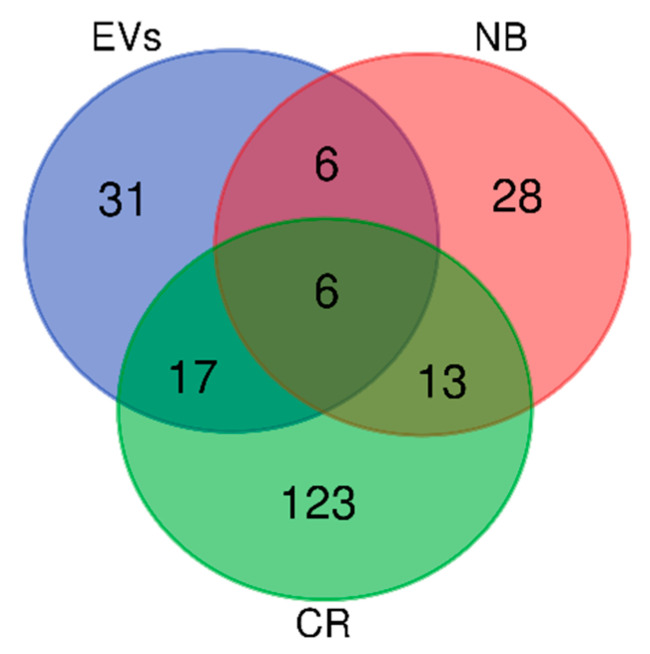
Venn diagram of all proteins identified: 31 were only Extracellular Vesicles proteins, 28 only non-brain proteins and 123 only Cancer Related proteins; 6 were both EVs and NB proteins, 13 both CR and NB proteins, 17 both EVs and CR proteins. Finally, 6 were identified as EVs, NB and CR proteins.

Moreover, it was interesting to investigate how many elements identified in GBM share all the three sub-proteomes classification. Grouping analysis of all data results in 31, 28 and 123 elements exclusive to the EVs, NB and CR sub-proteome, respectively, and 6 elements common to all three protein groups (Figure 16). Tables S4–S7 in Supplementary Materials list all these elements and their zone of identification.

From these data particular attention was paid to the six proteins sharing all sub-proteomes classification and therefore could be the most significant in molecular depiction of GBM disease and its rate of diffusion and relapse (Table 11).

Some of these elements have been already studied and correlated to GBM, while others have never been investigated. It is interesting to note that all of them were identified in all the zones analyzed, i.e., both in the tumor (CORE and A+) and in the healthy (A−) zone of ND- and R-GBM pools, with the exception of complement factor H-related protein 1, not identified in ND-GBM, and of inter-alpha-trypsin inhibitor heavy chain H4 protein, that in ND-GBM was not identified in the tumor core.

**Figure 17:**
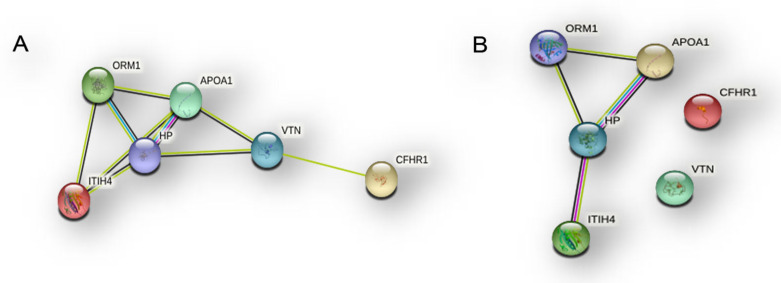
STRING analyses of the six elements common to the three sub-proteomes, i.e., NB, CR and EVs proteins with (**A**) medium confidence and (**B**) high confidence.

STRING tool analysis showed that these proteins are enclosed in an interaction network by setting either the medium (Figure 17A) or the high (Figure 17B) analysis confidence, thus resulting as being functionally interconnected.

**Figure 18:**
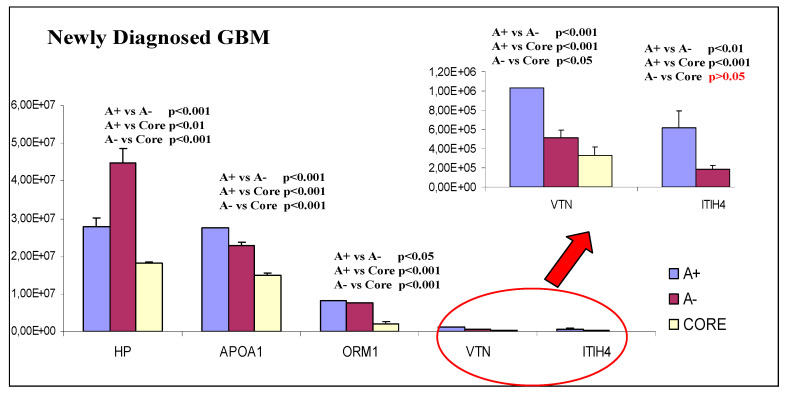
Quantitative trend of the five proteins common to NB, CR and EVs sub-proteome identified in ND-GBM pool, with enlargement to appreciate the trends of VTN and ITIH4, and with *p*-value expression.

**Figure 19:**
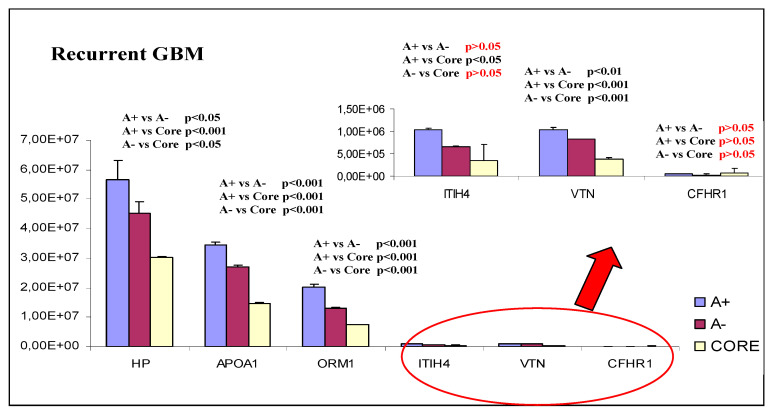
Quantitative trend of the six proteins common to NB, CR and EVs sub-proteome identified in R-GBM pool, with enlargement to appreciate the trends of ITIH4, VTN and CFHR, and with *p*-value expression.

These six very interesting proteins were also analyzed by label free relative quantization to investigate eventual significant statistical differences between ND- and R-GBM tumor diagnoses and/or the different zones. By observing the graphs in Figure 18 and Figure 19, it is possible to note that all proteins in both pools show the same general trend, with levels decreasing from A+ to A− and CORE zones, with statistical significance and few exceptions.

Only two of these proteins did not follow this trend: the Haptoglobin (HP) in the ND-GBM pool (Figure 18) and the complement factor H-related protein 1 (CFHR1) in the R-GBM pool (Figure 19).

CFHR1 showed lower levels in the R-GBM pool in the A− zone with respect to the CORE and to the A+ zone; however, these differences were not statistically significant. The HP protein showed instead the opposite behavior of CFHR1, exhibiting in the ND-GBM pool statistically significant higher levels in the A− zone.

The biological role and significance of the six proteins that can be classified in all three sub-proteomes studied are discussed below in separate paragraphs.

#### 4.4.1. Vitronectin (VTN)

VTN is an element of extracellular matrix. Its expression in GBM is a measure of the tumor’s stage. VTN functions in cancer disease consist in allowing the tumor migrations, conferring chemoresistance and inhibiting topoisomerases (responsible of the apoptosis), raising the survival of tumor cells [50].

#### 4.4.2. Apolipoprotein A-1 (APOA1)

APOA1 is a member of a large family of proteins that bind and transport lipids to tissues for use and metabolization. In tumor disease the expression of this protein is modulated: in some cases, it results as being overexpressed, in others cases the opposite occurs [51]. Relative to the involvement of APOA1 in GBM disease, no studies have been reported yet, to the best of our knowledge. The unique apolipoprotein that seems to be involved in GBM disease is the Apolipoprotein E (APOE), which promotes tumor growth and metastasis by inhibiting the immune system [51]. In the present investigation we found APOE in the lists of Cancer Related and Extracellular Vesicles proteins, in both Newly Diagnosed and Recurrent pools and in all three zones.

#### 4.4.3. Haptoglobin (HP)

HP is a glycoprotein of the plasma that is involved in anti-inflammatory processes, in prophylaxis of oxidative damage and in tissue protection. Its primarily role is to bind hemoglobin (Hb) to increase its affinity with the CD163 receptor on hepatocytes, monocytes and macrophages [52]. In this way, HP removes free Hb by the plasma, preventing the production of radical species of oxygen catalyzed by heme. Other HP functions are inhibition of prostaglandin synthesis, support of the angiogenesis and modulation of the immune system [52]. This last function is very important in tumor disease because it was demonstrated, in a study on lung cancer, that HP can protect the tumor cells from attack by the immune system [53]. In breast cancers it was demonstrated that HP can modulate the cellular cycle and apoptosis in cancer cells [54]. For all these reasons, high levels of HP are associated to poor prognosis in several cancer types, such as ovarian, colorectal, pancreatic, breast and hepatocellular carcinomas [55]. Its high expression in blood has been correlated also with glioblastoma multiforme. Particularly the unprocessed pre-HP, called zonulin, was identified in this kind of brain cancer [56]. It was also demonstrated in studies in vitro on GBM that HP increases cell migration [52]. This evidence, joined to the highest presence of this protein in the A− zone rather than the A+ zone in ND-GBM (Figure 18), could correlate this protein to the GBM tumor invasiveness.

#### 4.4.4. Complement Factor H-Related Protein 1 (CFHR1)

CFHR1 is the truncated form of complement factor H (that is present in the list of CR and EVs proteins), an inhibitor of the complement alternative pathway. CFHR1 preserves the same function of the untruncated form of complement factor H, i.e., preventing cellular death by lysis process. It was reported that human H2 glioblastoma cells synthesize high levels of CFHR1, so the protein can be hypothesized to be involved in one of the mechanisms used to avoid cellular death, typical of tumors [40]. Being identified only in R-GBM, this protein could be a candidate to distinguish GBMs of new diagnosis from the tumor relapse.

#### 4.4.5. Inter-Alpha-Trypsin Inhibitor Heavy Chain H4 (ITIH4)

ITIH4 is a liver-specific protein involved in the inflammatory reaction to a trauma [31] and reported in a hepatocellular carcinoma study [57]. This protein was never correlated to GBM disease, to the best of our knowledge, therefore a deeper investigation would be interesting. Its identification in ND-GBM only in the tumor periphery could be applied to distinguish the ND- from R-GBM CORE.

#### 4.4.6. Alpha-1-Acid Glycoprotein 1(ORM1)

ORM1 is a modulator of the immune system during the acute phase of inflammation. Another role of this protein is the transportation of several proteins in blood. A correlation with GBM was reported in late 1985 by Matsuura and Nakazawa reporting an overexpression of this protein in patients affected by glioblastoma multiforme. They hypothesized that ORM1 inhibits T and B cell proliferation [58].

### 4.5. Proteins Detected in the Healthy Zone of ND-GBMs and in the CORE of R-GBMs

Finally, considering the total proteins identified in ND and R-GBM pools and classified in NB, CR and EVs sub-proteomes, a particular focus was due to the proteins that were in common between the ND-GBM A− zone and R-GBM tumor zone, to disclose protein elements possibly correlated to the onset of tumor relapse occurring at the same site of the primary tumor.

Table 12 lists the elements found only in the healthy zone (A−) of Newly Diagnosed GBM and their relative zone of identification in Recurrent GBM. In the last column their sub-proteome classification is also reported.

It is possible to note that all of them were detected in the tumor zone of R-GBM (CORE and A+ zones). Particular attention was paid to three of them: Histone H2A type 1-B/E (H2A1B), macrophage migration inhibitory factor (MIF) and Tropomyosin alpha-1 chain (TPM1), which are present only in the tumor zones of R-GBM, in particular in CORE. This evidence is very important because it could be supposed that these proteins, two CR and one NB, can participate in the molecular processes leading to the occurrence of the relapse phenomena.

**Figure 20:**
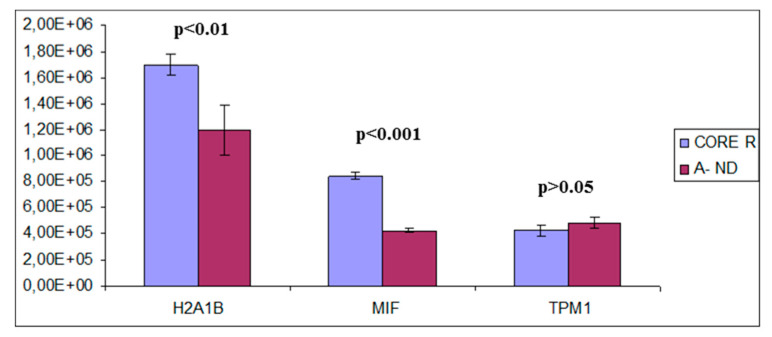
Quantitative analysis of H2A1B, MIF and TPM1, proteins detected in A− ND-GBM and in CORE R-GBM. The statistical significant differences are additionally reported (*p*-value, *p*).

A quantitative analysis of these three proteins, illustrated in Figure 20, showed higher levels of H2A1B and MIF in R-GMB CORE with respect to A− ND-GBM, with a *p* value that confirmed a statistical relevance of these differences.

On the contrary, TPM1 showed not statistically significant different levels (*p* > 0.05) between the R-GBM CORE and the ND-GBM A− zone.

While histones were already discussed above (Section 4.1. Proteins not Detected in Brain (NB)), a discussion on MIF and TPM1 is reported below.

MIF is a pro-inflammatory cytokine synthesized by numerous kinds of cells. In GBM, a correlation was seen between high levels of MIF and tumor recurrence, which is confirmed also by our data. As principal function of this protein, a lot of studies demonstrated an important and significant role in angiogenesis. MIF inhibitors were developed as a therapeutic strategy. Even if in vitro they gave good results, in vivo more studies are still necessaries [59].

TPM1 is an actin-binding cytoskeletal protein with a recognized tumor suppressor function in a lot of cancers. It was demonstrated that TPM1 suppresses tumor growth and proliferation as well as angiogenesis in renal carcinoma [60]. Concerning GBM, this protein is often found downregulated in other studies, and one of them demonstrated that this downregulation is correlated to the resistance of tumor cells to radiotherapy; however, the comprehension of this mechanism needs more studies [61].

The evaluation of H2A1B, MIF and TPM1 levels in other biological fluid of low invasiveness collection, such as blood or saliva, could be a possible alarm bell for an early diagnosis of GBM recurrence to be investigated in a future study.

## 5. Conclusions

The present investigation tried to shed light on some still unexplored territories of the GBM proteome. By applying a computational approach to the experimental proteomic data of GBM CUSA pools analysis collected from different tumor zones, it was possible to investigate and classify three different sub-proteomes in GBM, i.e., Non Brain proteins, Cancer Related proteins and Extracellular Vesicles proteins, based on the Human Protein Atlas [5] classification. These specific classes of proteins, due to their involvement in oncogenesis and tumor diffusion, were particularly interesting to investigate in the purpose to understand the molecular mechanisms at the basis of the high aggressiveness and recurrence rate of GBM tumors. The finding of proteins non-classified as brain proteins, together with evidencing a possible Blood Brain Barrier disruption and bleeding, frequently occurring in brain tumor disease, open the ability to investigate unexplored mechanisms related to the onset and progression of the tumor. Moreover, the results showed protein elements of these specific protein classes that could be potential candidate biomarkers to distinguish ND- from R-GBM and the different zones, i.e., tumor core and periphery, directing to further investigations and validation studies. The finding of these classes of proteins in the A− tumor periphery, the so called “healthy zone”, in addition to the tumor zone, seems to reveal a possible tumor infiltration in the healthy zone that is undetectable by surgery fluorescence. As future perspective, single cell proteomics could be the tool of choice to ascertain and localize tumor cell infiltration in the brain parenchyma as well as to profile precursor tumor cells.

In addition to the demand to identify specific histological features able to distinguish healthy brain tissue at the tumor periphery in addition to protoporphyrin IX fluorescence, the future perspective of this study is directed to the application of iKnife mass spectrometry to guide tumor resection by profiling selective markers at the tumor margins. Moreover, another standpoint will be to investigate proteomic data correlation in biological fluid, such as blood or saliva, to confirm and validate the results by using samples of low invasiveness collection and easily available for normal control sample of reference and to disclose potential biomarkers for clinical applications. Likewise, the reclassification of these data by single patient analysis rather than specimen pools could help in the evaluation of the individual variations and to direct the research to a personalized medicine approach.

## Figures and Tables

**Table 1:** List of 4 NB proteins exclusively identified in ND-GBM pool.

Uniprot	Protein Name	ND Zones
Q96PD5	N-acetylmuramoyl-L-alanine amidase	A+
Q562R1	Beta-actin-like protein 2	A+
P35527	Keratin, type I cytoskeletal 9	A+
P08697	Alpha-2-antiplasmin	A+

**Table 2:** List of 6 NB proteins exclusively identified in R-GBM pool.

Uniprot	Protein Name	R Zones
P35542	Serum amyloid A-4 protein	A+
Q06033	Inter-alpha-trypsin inhibitor heavy chain H3	A+
P0DJI8	Serum amyloid A-1 protein	A+ CORE
Q8WZ42	Titin	A+
Q03591	Complement factor H-related protein 1	A+ CORE A−
P02748	Complement component C9	A+

**Table 3:** Description of the 5 NB non-blood proteins found in Newly Diagnosed and Recurrent GBMs pools.

Uniprot	Protein Name	ND Zones	R Zones
P23527	Histone H2B type 1-O	CORE A−	CORE
Q96KK5	Histone H2A type 1-H	CORE A+ A−	CORE A+ A−
P62805	Histone H4	A+	A+
P04908	Histone H2A type 1-B/E	A−	CORE
P0DJI8	Serum amyloid A-1 protein		A+ CORE

**Table 4:** List of the 15 CR proteins exclusively identified in ND-GBM pool.

Uniprot	Protein Name	ND Zones
P00533	Epidermal growth factor receptor	A+
Q15836	Vesicle-associated membrane protein 3	A+
P20073	Annexin A7	A+
Q15056	Eukaryotic translation initiation factor 4H	A+
Q15365	Poly(rC)-binding protein 1	A+
P13987	CD59 glycoprotein	CORE
P23297	Protein S100-A1	A+
P07355	Annexin A2	A+
P36222	Chitinase-3-like protein 1	A+
P07951	Tropomyosin beta chain	CORE
P26447	Protein S100-A4	A+
P17931	Galectin-3	A+
P27824	Calnexin	A+
P54727	UV excision repair protein RAD23 homolog B	A+
P13010	X-ray repair cross-complementing protein 5	A+

**Table 5:** List of the 16 CR proteins only detected in R-GBM pool.

Uniprot	Protein Name	R Zones
P31751	RAC-beta serine/threonine-protein kinase	A−
Q00577	Transcriptional activator protein Pur-alpha	A−
P02545	Prelamin-A/C	CORE A−
P38159	RNA-binding motif protein, X chromosome	CORE
P67809	Nuclease-sensitive element-binding protein 1	CORE
Q99961	Endophilin-A2	CORE
P19367	Hexokinase-1	A−
Q02952	A-kinase anchor protein 12	CORE A−
P54652	Heat shock-related 70 kDa protein 2	A+ A−
Q15843	NEDD8	A+ A−
Q03591	Complement factor H-related protein 1	CORE A+ A−
P63000	Ras-related C3 botulinum toxin substrate 1	CORE A−
P84103	Serine/arginine-rich splicing factor 3	CORE
P68402	Platelet-activating factor acetylhydrolase IB subunit beta	CORE A+ A−
P63167	Dynein light chain 1, cytoplasmic	A+ A−
P61769	Beta-2-microglobulin	A−

**Table 6:** List of the 6 EVs proteins exclusive to Newly Diagnosed GBM pool.

Uniprot	Protein Name	ND Zones
P36222	Chitinase-3-like protein 1	A+
Q9GZV4	Eukaryotic translation initiation factor 5A-2	A−
P27105	Erythrocyte band 7 integral membrane protein	A+ A−
P13987	CD59 glycoprotein	CORE
Q9H4G4	Golgi-associated plant pathogenesis-related protein 1	A+
Q15181	Inorganic pyrophosphatase	A+

**Table 7:** List of 14 EVs proteins typical of Recurrent GBM pool.

Uniprot	Protein Name	R Zones
P31751	RAC-beta serine/threonine-protein kinase	A−
P67809	Nuclease-sensitive element-binding protein 1	CORE
P47756	F-actin-capping protein subunit beta	A− CORE
Q6PUV4	Complexin-2	CORE
Q03591	Complement factor H-related protein 1	CORE A+ A−
Q9BY11	Protein kinase C and casein kinase substrate in neurons protein 1	CORE A+ A−
P25398	40S ribosomal protein S12	CORE
P09497	Clathrin light chain B	CORE
Q8N6N7	Acyl-CoA-binding domain-containing protein 7	CORE
P54652	Heat shock-related 70 kDa protein 2	A+ A−
P00739	Haptoglobin-related protein	A+
Q9UHG2	ProSAAS	CORE
O14745	Na(+)/H(+) exchange regulatory cofactor NHE-RF1	A− CORE
Q9NRV9	Heme-binding protein 1	A−

**Table 8:** Cancer Related proteins exclusive to ND-GBM and their role in cancer disease.

Gene Name	ND Zones	Protein Function and References	GBM Identification
EGFR	A+	Promote tumor survival, proliferation and invasion [17]	Yes
VAMP3	A+	Support EGFR in its functions [18]	Yes
ANXA7	A+	Tumor suppressor, downregulated in cancer [19]	Yes
IF4H	A+	Associated to poor prognosis; promote the helicases activity [20]	Yes
PCBP1	A+	Inhibitor of tumor onset and metastasis; downregulated in cancers [21]	No
CD59	CORE	Overexpressed; regulate the functions and the infiltration of immune cells in tumor [22]	No
S10A1	A+	Anti-apoptotic [23]	No
ANXA2	A+	Over-represented in higher grade GBM; stimulate angiogenesis, proliferation and invasiveness [24]	Yes
CH3L1	A+	Promoting of tumor growth, proliferation, invasion and metastasis; overexpressed in cancers [25]	Yes
TPM2	CORE	Modulate invasion and migration [26]	No
S10A4	A+	Overexpressed in cancers; correlated with the occurrence of metastasis and associated to poor prognosis [27]	No
LEG3	A+	Induce endothelial cell differentiation [28]	Yes
CALX	A+	Inhibits the infiltration and the functions of T cells [29]	No
RAD23B	A+	Involved in genome nucleotide excision repair [30]	No
XRCC5	A+	Mutagenic factor [31]	No

**Table 9:** Cancer Related proteins exclusive to R-GBM and their role in cancer disease.

Gene Name	R Zones	Protein Function and References	GBM Identification
AKT2	A−	Involved in metabolism, cellular growth and survival, angiogenesis [31]	No
PURA	A−	Transcriptional activator protein overexpressed in GBM, where interact with tumor associated genes [32]	Yes
LMNA	CORE A−	Possible biomarkers of aggressiveness; associated to poor prognosis [33]	Yes
RBMX	CORE	All RNA binding proteins are overexpressed in GBM and associated with poor prognosis, but RBMX protein has never been detected [34]	No
YBOX1	CORE	Like LMNA, it is a transcriptional activator protein overexpressed in GBM, where interact with tumor associated genes [32]	Yes
SH3G1	CORE	Involved in oncogenesis; its expression is directly proportional to tumor progression [35]	Yes
HXK1	A−	Increased glycolysis and oncogenesis; its isoform HXK2 has been found in GBM [36]	No
AKA12	CORE A−	Downregulated in GBM; tumor suppressor [37]	Yes
HSP72	A+ A−	Chaperones; inhibits proliferation of T cells [38]	Yes
NEDD8	A+ A−	Inhibits the immune activity; it is a target for therapy under study [39]	Yes
CFHR1	CORE A+ A−	Inhibitor of complement alternative pathway [40]	Yes
RAC1	CORE A−	Promote radio resistance in GBM [41]	Yes
SRSF3	CORE	Upregulated; associated to tumor progression and poor prognosis [42]	Yes
PA1B2	CORE A+ A−	Involved in migration, invasion and metastasis [43]	No
DYL1	A+ A−	Overexpressed; promote proliferation and invasion [44]	Yes
B2MG	A−	Correlated with malignancy and immune signatures [45]	Yes

**Table 10:** EVs proteins correlated with GBM and other brain tumors.

Uniprot	Protein Name	ND Zones	R Zones
P04406	Glyceraldehyde-3-phosphate dehydrogenase	CORE A+ A−	CORE A+ A−
P07602	Prosaposin	A+	CORE A+ A−
P36222	Chitinase-3-like protein 1	A+	Not detected

**Table 11:** List of the six common proteins to all three sub-proteomes.

Uniprot	Protein Name	ND Zones	R Zones
P04004	Vitronectin	CORE A+ A−	CORE A+ A−
P02647	Apolipoprotein A-I	CORE A+ A−	CORE A+ A−
P00738	Haptoglobin	CORE A+ A−	CORE A+ A−
Q03591	Complement factor H-related protein 1		CORE A+ A−
Q14624	Inter-alpha-trypsin inhibitor heavy chain H4	A+ A−	CORE A+ A−
P02763	Alpha-1-acid glycoprotein 1	CORE A+ A−	CORE A+ A−

**Table 12:** List of protein elements identified in the A− zone of ND-GBM pool and identified in R-GBM and their zone of identification in R-GBM.

Uniprot	Protein Name	R-GBM Zones	Sub-Proteome
P07451	Carbonic anhydrase 3	CORE A+ A−	NB
P04908	Histone H2A type 1-B/E	CORE	NB
P13671	Complement component C6	A+	NB/CR
P14174	Macrophage migration inhibitory factor	CORE A+	CR
P20810	Calpastatin	A+	CR
P09493	Tropomyosin alpha-1 chain	CORE	CR
P05109	Protein S100-A8	A+ A−	CR
P07998	Ribonuclease pancreatic	CORE A+ A−	EVs

## Data Availability

Data is contained within the article or supplementary material. The protein identification data investigated in this study are available in [1].

## Supplementary Materials

The following supporting information can be downloaded at: https://www.mdpi.com/article/10.3390/ijms23042058/s1.
