# Supplementary material for: Investigating Glioblastoma Multiforme Sub-Proteomes: A Computational Study of CUSA Fluid Proteomic Data
Source: Int J Mol Sci. 2022 Feb 12;23(4):2058. doi: 10.3390/ijms23042058 (PMC8879425; doi:10.3390/ijms23042058)
Supplement: Supplementary file 1 [file ijms-23-02058-s001.zip › ijms-1546271-supplementary.pdf]

Investigating Glioblastoma Multiforme Sub-Proteomes: A Computational Study of CUSA Fluid Proteomic Data

SUPPLEMENTARY MATERIALS

Table S1. *List of 43 NB proteins identified in both ND- and R-GBMs pools.*

Uniprot	Protein Name	ND Zones	R Zones
P02656	Apolipoprotein C-III	CORE A-	CORE A- A+
P06727	Apolipoprotein A-IV	CORE A- A+	CORE A- A+
P00747	Plasminogen	CORE A- A+	CORE A- A+
P02743	Serum amyloid P-component	A+	A-
P27169	Serum paraoxonase/arylesterase 1	A+	A+ A-
P02790	Hemopexin	CORE A+ A-	CORE A+ A-
P07451	Carbonic anhydrase 3	A-	CORE A+ A-
P02750	Leucine-rich alpha-2-glycoprotein	CORE A+	CORE A-
P02675	Fibrinogen beta chain	A+ A-	CORE A+ A-
P01042	Kininogen-1	CORE A+ A-	CORE A+ A-
Q96KK5	Histone H2A type 1-H	CORE A+ A-	CORE A+ A-
P59665	Neutrophil defensin 1	CORE A+ A-	A+ A-
P02652	Apolipoprotein A-II	CORE A+ A-	CORE A+ A-
P02768	Serum albumin	CORE A+ A-	CORE A+ A-
P02749	Beta-2-glycoprotein 1	CORE A+ A-	CORE A+ A-
P02765	Alpha-2-HS-glycoprotein	CORE A+ A-	CORE A+ A-
P04217	Alpha-1B-glycoprotein	CORE A+ A-	CORE A+ A-
P00738	Haptoglobin	CORE A+ A-	CORE A+ A-
P19652	Alpha-1-acid glycoprotein 2	CORE A+ A-	CORE A+ A-
P02671	Fibrinogen alpha chain	CORE A+ A-	CORE A+ A-
P02549	Spectrin alpha chain, erythrocytic 1	CORE A+ A-	CORE A+ A-
Q14624	Inter-alpha-trypsin inhibitor heavy chain H4	A+ A-	CORE A+ A-
P04004	Vitronectin	CORE A+ A-	CORE A+ A-
O43866	CD5 antigen-like	A+ A-	CORE A+ A-
P00748	Coagulation factor XII	A+	A+ A-
P02760	Protein AMBP	CORE A+ A-	CORE A+ A-
P02679	Fibrinogen gamma chain	A+ A-	CORE A+ A-
P04908	Histone H2A type 1-B/E	A-	CORE
P43652	Afamin	CORE A+ A-	CORE A+ A-
P04114	Apolipoprotein B-100	A+ A-	CORE A+ A-
P04264	Keratin, type II cytoskeletal 1	CORE A+	A-
P02774	Vitamin D-binding protein	CORE A+ A-	CORE A+ A-
P19827	Inter-alpha-trypsin inhibitor heavy chain H1	A+ A-	CORE A+ A-
P04003	C4b-binding protein alpha chain	A+ A-	CORE A+ A-
P62805	Histone H4	A+	A+
P68032	Actin, alpha cardiac muscle 1	CORE A-	CORE A+ A-
P00734	Prothrombin	CORE A+ A-	CORE A+ A-
P01008	Antithrombin-III	CORE A+ A-	CORE A+ A-
P23527	Histone H2B type 1-O	CORE A-	CORE
P02647	Apolipoprotein A-I	CORE A+ A-	CORE A+ A-
P04196	Histidine-rich glycoprotein	CORE A+ A-	CORE A+ A-
P02763	Alpha-1-acid glycoprotein 1	CORE A+ A-	CORE A+ A-
P13671	Complement component C6	A-	A+

Table S2. *List of the 128 common ND- and R-GBMs Cancer Related proteins.*

Uniprot	Protein Name	ND Zones	R Zones
P09211	Glutathione S-transferase	A+	A+ A-
P02656	Apolipoprotein C-III	A- CORE	CORE A+ A-
P27169	Serum paraoxonase/arylesterase	A+	A+ A-
P60201	Myelin proteolipid protein	A+	A+ A-
P02675	Fibrinogen beta chain	A+ A-	CORE A+ A-
P53396	ATP-citrate synthase	A+	A+ A-
P02794	Ferritin heavy chain	A+ A-	CORE A+ A-
P13639	Elongation factor 2	A+	A+ A-
P16949	Stathmin	A- CORE	CORE A+ A-
P14174	Macrophage migration inhibitory factor	A-	CORE A+
P14618	Pyruvate kinase PKM	CORE A+ A-	CORE A+ A-
P04004	Vitronectin	CORE A+ A-	CORE A+ A-
P68104	Elongation factor 1-alpha 1	CORE A+ A-	CORE A+ A-
P21266	Glutathione S-transferase Mu 3	A+	A-
P06753	Tropomyosin alpha-3 chain	A+ A-	CORE A+ A-
P06748	Nucleophosmin	CORE A+ A-	CORE A-
P61088	Ubiquitin-conjugating enzyme E2 N	A+	A- A+
P54725	UV excision repair protein RAD23 homolog A	A+	A-
Q00610	Clathrin heavy chain 1	A+	A- A+
P49418	Amphiphysin	A+	A- A+
P00450	Ceruloplasmin	CORE A+ A-	CORE A+ A-
P02751	Fibronectin	CORE A+ A-	CORE A+ A-
P08238	Heat shock protein HSP 90-beta	CORE A+ A-	CORE A+ A-
P61604	10 kDa heat shock protein, mitochondrial	CORE A+ A-	CORE A+ A-
P02647	Apolipoprotein A-I	CORE A+ A-	CORE A+ A-
P16070	CD44 antigen	CORE	CORE
Q01469	Fatty acid-binding protein, epidermal	CORE A+ A-	CORE A+
P04075	Fructose-bisphosphate aldolase A	CORE A+ A-	CORE A+ A-
Q15149	Plectin	A+ A-	CORE A+ A-
P60174	Triosephosphate isomerase	CORE A+ A-	CORE A+ A-
P05023	Sodium/potassium-transporting ATPase subunit alpha-1	A+ A-	CORE A-
P32119	Peroxiredoxin-2	CORE A+ A-	CORE A+ A-
P31939	Bifunctional purine biosynthesis protein PURH	A+	A-
P11142	Heat shock cognate 71 kDa protein	CORE A+ A-	CORE A+ A-
P22626	Heterogeneous nuclear ribonucleoproteins A2/B1	CORE A+ A-	CORE A+ A-
P15311	Ezrin	A+ A-	CORE
P02649	Apolipoprotein E	CORE A+ A-	CORE A+ A-
P00738	Haptoglobin	CORE A+ A-	CORE A+ A-
P02792	Ferritin light chain	A+ A-	CORE A+ A-
P01034	Cystatin-C	A+ A-	A+ A-
P04179	Superoxide dismutase [Mn], mitochondrial	A+	CORE A+ A-
P48735	Isocitrate dehydrogenase [NADP], mitochondrial	A+	A+ A-
P01023	Alpha-2-macroglobulin	CORE A+ A-	CORE A+ A-
P06733	Alpha-enolase	CORE A+ A-	CORE A+ A-
P02671	Fibrinogen alpha chain	CORE A+ A-	CORE A+ A-
P09972	Fructose-bisphosphate aldolase C	CORE A+ A-	CORE A+ A-
P02654	Apolipoprotein C-I	A+	A+ A-
P00338	L-lactate dehydrogenase A chain	A+	CORE A+ A-
P09104	Gamma-enolase	A+ A-	CORE A+ A-
P10412	Histone H1.4	CORE	CORE
P04040	Catalase	CORE A+ A-	CORE A+ A-

P00441	Superoxide dismutase [Cu-Zn]	CORE A+ A-	CORE A+ A-
P25311	Zinc-alpha-2-glycoprotein	CORE A+ A-	CORE A+ A-
P06396	Gelsolin	A+ A-	CORE A+ A-
P62258	14-3-3 protein epsilon	A+	A+ A-
P07339	Cathepsin D	A+	A-
P06744	Glucose-6-phosphate isomerase	A+	CORE A+ A-
P02787	Serotransferrin	CORE A+ A-	CORE A+ A-
P06702	Protein S100-A9	A+ A-	CORE A+ A-
P17174	Aspartate aminotransferase, cytoplasmic	A+ A-	A+ A-
P14625	Endoplasmin	A+	A-
P10909	Clusterin	CORE A+ A-	CORE A+ A-
P62805	Histone H4	A+	A+
P10809	60 kDa heat shock protein, mitochondrial	CORE A+ A-	CORE A+ A-
P22314	Ubiquitin-like modifier-activating enzyme 1	A+	A+ A-
P04080	Cystatin-B	A+	CORE A+ A-
P13671	Complement component C6	A-	A+
Q15019	Septin-2	A+ A-	CORE A+ A-
P00747	Plasminogen	CORE A+ A-	CORE A+ A-
Q92597	Protein NDRG1	A+	CORE A-
P34932	Heat shock 70 kDa protein 4	A+	A+ A-
P02766	Transthyretin	CORE A+ A-	CORE A+ A-
P08603	Complement factor H	CORE A+ A-	CORE A+ A-
P10451	Osteopontin	CORE	A-
P01011	Alpha-1-antichymotrypsin	A+ A-	A+ A-
P43003	Excitatory amino acid transporter 1	A+ A-	CORE A-
P35579	Myosin-9	A+ A-	CORE A+ A-
P02768	Serum albumin	CORE A+ A-	CORE A+ A-
P04083	Annexin A1	A+	CORE A+ A-
Q14624	Inter-alpha-trypsin inhibitor heavy chain H4	A+ A-	CORE A+ A-
Q04760	Lactoylglutathione lyase	A+	CORE A+ A-
O00299	Chloride intracellular channel protein 1	A+	A+
P02511	Alpha-crystallin B chain	CORE A+ A-	CORE A+ A-
P24821	Tenascin	A+ CORE	CORE
P36955	Pigment epithelium-derived factor	A+	A-
P01009	Alpha-1-antitrypsin	CORE A+ A-	CORE A+ A-
P30153	Serine/threonine-protein phosphatase 2A 65 kDa regulatory subunit A alpha isoform	A+	A-
P02679	Fibrinogen gamma chain	A+ A-	CORE A+ A-
P02753	Retinol-binding protein 4	A+	A+ A-
P02788	Lactotransferrin	CORE A+ A-	CORE A+ A-
P20810	Calpastatin	A-	A+
P21926	CD9 antigen	A+	A+
Q13526	Peptidyl-prolyl cis-trans isomerase NIMA-interacting 1	CORE A-	CORE A+ A-
P02763	Alpha-1-acid glycoprotein 1	CORE A+ A-	CORE A+ A-
Q14847	LIM and SH3 domain protein 1	A+	CORE
P27797	Calreticulin	A+	A-
P62873	Guanine nucleotide-binding protein G(I)/G(S)/G(T) subunit beta-1	A+ A-	CORE A+ A-
P67936	Tropomyosin alpha-4 chain	CORE A+ A-	CORE A+ A-
Q71U36	Tubulin alpha-1A chain	A+ A-	A+ A-
P59665	Neutrophil defensin 1	CORE A+ A-	A+ A-
Q01105	Protein SET	A+	A-
P07108	Acyl-CoA-binding protein	A+	A-
P02652	Apolipoprotein A-II	CORE A+ A-	CORE A+ A-

Q08380	Galectin-3-binding protein	A+	A+
P02765	Alpha-2-HS-glycoprotein	CORE A+ A-	CORE A+ A-
P09493	Tropomyosin alpha-1 chain	A-	CORE
P04792	Heat shock protein beta-1	CORE A+ A-	CORE A+ A-
P43490	Nicotinamide phosphoribosyltransferase	A+	CORE A+ A-
P05090	Apolipoprotein D	CORE A+ A-	CORE A+ A-
P05164	Myeloperoxidase	A+ A-	CORE
P40925	Malate dehydrogenase, cytoplasmic	CORE A+ A-	CORE A+ A-
P15531	Nucleoside diphosphate kinase A	A+	A+ A-
P00505	Aspartate aminotransferase, mitochondrial	A+	A+ A-
P07900	Heat shock protein HSP 90-alpha	CORE A+ A-	CORE A+ A-
Q16658	Fascin	A+ A-	CORE A+ A-
Q04917	14-3-3 protein eta	A+	CORE A+ A-
P35637	RNA-binding protein FUS	A+	CORE
P63241	Eukaryotic translation initiation factor 5A-1	A+	CORE A+ A-
P00734	Prothrombin	CORE A+ A-	CORE A+ A-
P11166	Solute carrier family 2, facilitated glucose transporter member 1	A+ A-	A+
P07954	Fumarate hydratase, mitochondrial	A+	A+
P31946	14-3-3 protein beta/alpha	CORE A+ A-	CORE A+ A-
P05109	Protein S100-A8	A-	A+ A-
P13591	Neural cell adhesion molecule 1	A+ A-	A+ A-
P12277	Creatine kinase B-type	CORE A+ A-	CORE A+ A-
P26038	Moesin	CORE A+ A-	CORE A+ A-
P61586	Transforming protein RhoA	A+	A+ A-
P22392	Nucleoside diphosphate kinase B	A+ A-	A-

Table S3. *List of the 40 EVs proteins common to ND- and R-GBMs pools.*

Uniprot	Protein name	ND zones	R zones
P23471	Receptor-type tyrosine-protein phosphatase zeta	A+ A-	CORE A+ A-
P06727	Apolipoprotein A-IV	CORE A+ A-	CORE A+ A-
P02750	Leucine-rich alpha-2-glycoprotein	CORE A+	CORE A-
P60174	Triosephosphate isomerase	CORE A+ A-	CORE A+ A-
P50897	Palmitoyl-protein thioesterase 1	A+	A+ A-
P04406	Glyceraldehyde-3-phosphate dehydrogenase	CORE A+ A-	CORE A+ A-
P62328	Thymosin beta-4	A+ A-	CORE A+ A-
P0DMV8	Heat shock 70 kDa protein 1A	A+ A-	CORE A+ A-
P07998	Ribonuclease pancreatic	A-	CORE A+ A-
Q13813	Spectrin alpha chain, non-erythrocytic 1	CORE A+ A-	CORE A+ A-
P38606	V-type proton ATPase catalytic subunit A	A+	A+ A-
P21281	V-type proton ATPase subunit B	A+	CORE A+ A-
P08603	Complement factor H	CORE A+ A-	CORE A+ A-
P11142	Heat shock cognate 71 kDa protein	CORE A+ A-	CORE A+ A-
P30043	Flavin reductase (NADPH)	A+ A-	CORE A+ A-
P21796	Voltage-dependent anion-selective channel protein 1	A+	A+ A-
P02649	Apolipoprotein E	CORE A+ A-	CORE A+ A-
P61266	Syntaxin-1B	A+	A+ A-
P00738	Haptoglobin	CORE A+ A-	CORE A+ A-
P01034	Cystatin-C	A+ A-	A+ A-
P00751	Complement factor B	CORE A+ A-	CORE A+ A-
P13611	Versican core protein	CORE A+ A-	CORE A+ A-
P19652	Alpha-1-acid glycoprotein 2	CORE A+ A-	CORE A+ A-

P05164	Myeloperoxidase	A+ A-	CORE
Q14624	Inter-alpha-trypsin inhibitor heavy chain H4	A+ A-	CORE A+ A-
P09972	Fructose-bisphosphate aldolase C	CORE A+ A-	CORE A+ A-
P00338	L-lactate dehydrogenase A chain	A+	CORE A+ A-
P04004	Vitronectin	CORE A+ A-	CORE A+ A-
P04040	Catalase	CORE A+ A-	CORE A+ A-
P0DP23	Calmodulin-1	A+ A-	CORE A+ A-
P02760	Protein AMBP	CORE A+ A-	CORE A+ A-
P01009	Alpha-1-antitrypsin	CORE A+ A-	CORE A+ A-
Q00610	Clathrin heavy chain 1	A+	A+ A-
P60880	Synaptosomal-associated protein 25	A+ A-	CORE A+ A-
P25311	Zinc-alpha-2-glycoprotein	CORE A+ A-	CORE A+ A-
P04114	Apolipoprotein B-100	A+ A-	CORE A+ A-
P19827	Inter-alpha-trypsin inhibitor heavy chain H1	A+ A-	CORE A+ A-
P07602	Prosaposin	A+	CORE A+ A-
P02763	Alpha-1-acid glycoprotein 1	CORE A+ A-	CORE A+ A-
P02647	Apolipoprotein A-I	CORE A+ A-	CORE A+ A-

Table S4. *List of the six common proteins to all three sub proteomes.*

Uniprot	Protein Name	ND Zones	R Zones
P04004	Vitronectin	CORE A+ A-	CORE A+ A-
P02647	Apolipoprotein A-I	CORE A+ A-	CORE A+ A-
P00738	Haptoglobin	CORE A+ A-	CORE A+ A-
Q03591	Complement factor H-related protein 1		CORE A+ A-
Q14624	Inter-alpha-trypsin inhibitor heavy chain H4	A+ A-	CORE A+ A-
P02763	Alpha-1-acid glycoprotein 1	CORE A+ A-	CORE A+ A-

Table S5. *List of proteins that are both Cancer Related and Non Brain.*

Uniprot	Protein Name	ND Zones	R Zones
P02656	Apolipoprotein C-III	CORE A-	CORE A- A+
P27169	Serum paraoxonase/arylesterase 1	A+	A+ A-
P02675	Fibrinogen beta chain	A+ A-	CORE A+ A-
P02671	Fibrinogen alpha chain	CORE A+ A-	CORE A+ A-
P62805	Histone H4	A+	A+
P13671	Complement component C6	A-	A+
P00747	Plasminogen	CORE A- A+	CORE A- A+
P02768	Serum albumin	CORE A+ A-	CORE A+ A-
P02679	Fibrinogen gamma chain	A+ A-	CORE A+ A-
P59665	Neutrophil defensin 1	CORE A+ A-	A+ A-
P02652	Apolipoprotein A-II	CORE A+ A-	CORE A+ A-
P02765	Alpha-2-HS-glycoprotein	CORE A+ A-	CORE A+ A-
P00734	Prothrombin	CORE A+ A-	CORE A+ A-

Table S6. *List of elements that are both Cancer Related and Extracellular Vesicles proteins.*

Uniprot	Protein Name	ND Zones	R Zones
P31751	RAC-beta serine/threonine-protein kinase		A-
Q00610	Clathrin heavy chain 1	A+	A+ A-
P60174	Triosephosphate isomerase	CORE A+ A-	CORE A+ A-
P67809	Nuclease-sensitive element-binding protein 1		CORE
P11142	Heat shock cognate 71 kDa protein	CORE A+ A-	CORE A+ A-

P02649	Apolipoprotein E	CORE A+ A-	CORE A+ A-
P13987	CD59 glycoprotein	CORE	
P01034	Cystatin-C	A+ A-	A+ A-
P09972	Fructose-bisphosphate aldolase C	CORE A+ A-	CORE A+ A-
P00338	L-lactate dehydrogenase A chain	A+	CORE A+ A-
P04040	Catalase	CORE A+ A-	CORE A+ A-
P25311	Zinc-alpha-2-glycoprotein	CORE A+ A-	CORE A+ A-
P54652	Heat shock-related 70 kDa protein 2		A+ A-
P08603	Complement factor H	CORE A+ A-	CORE A+ A-
P36222	Chitinase-3-like protein 1	A+	
P01009	Alpha-1-antitrypsin	CORE A+ A-	CORE A+ A-
P05164	Myeloperoxidase	A+ A-	CORE

Table S7. *List of elements that are both Extracellular Vesicles proteins and Not detected in Brain.*

Uniprot	Protein Name	ND Zones	R Zones
P19827	Inter-alpha-trypsin inhibitor heavy chain H1	A+ A-	CORE A+ A-
P06727	Apolipoprotein A-IV	CORE A+ A-	CORE A+ A-
P02750	Leucine-rich alpha-2-glycoprotein	CORE A+	CORE A-
P19652	Alpha-1-acid glycoprotein 2	CORE A+ A-	CORE A+ A-
P02760	Protein AMBP	CORE A+ A-	CORE A+ A-
P04114	Apolipoprotein B-100	A+ A-	CORE A+ A-
